# Evaluation of unclassified variants in the breast cancer susceptibility genes *BRCA1 *and *BRCA2 *using five methods: results from a population-based study of young breast cancer patients

**DOI:** 10.1186/bcr1865

**Published:** 2008-02-19

**Authors:** Eunjung Lee, Roberta McKean-Cowdin, Huiyan Ma, Zhengjia Chen, David Van Den Berg, Brian E Henderson, Leslie Bernstein, Giske Ursin

**Affiliations:** 1 Department of Preventive Medicine, Keck School of Medicine, University of Southern California, 1441 Eastlake Avenue, USC/Norris Comprehensive Cancer Center, Los Angeles, CA 90089-9175, USA; 2 Institute for Health Promotion and Disease Prevention Research, Keck School of Medicine, University of Southern California, Alhambra, CA 91803, USA; 3 Children's Oncology Group, Arcadia, CA 91066, USA; 4 City of Hope Comprehensive Cancer Center and Beckman Research Institute, Duarte, CA 91010, USA; 5 Department of Nutrition, University of Oslo, Norway

## Abstract

**Introduction:**

Efforts are ongoing to determine the significance of unclassified variants (UVs) in the breast cancer susceptibility genes *BRCA1*/*BRCA2*, but no study has systematically assessed whether women carrying the suspected deleterious UVs have characteristics commonly seen among women carrying known deleterious or disease-causing mutations in *BRCA1*/*BRCA2*.

**Methods:**

We sequenced *BRCA1*/*BRCA2 *in 1,469 population-based female breast cancer patients diagnosed between the ages of 20 and 49 years. We used existing literature to classify variants into known deleterious mutations, polymorphic variants, and UVs. The UVs were further classified as high risk or low risk based on five methods: allele frequency, Polyphen algorithm, sequence conservation, Grantham matrix scores, and a combination of the Grantham matrix score and sequence conservation. Furthermore, we examined whether patients who carry the variants classified as high risk using these methods have risk characteristics similar to patients with known deleterious *BRCA1*/*BRCA2 *mutations (early age at diagnosis, family history of breast cancer or ovarian cancer, and negative estrogen receptor/progesterone receptor).

**Results:**

We identified 262 distinct *BRCA1*/*BRCA2 *variants, including 147 UVs, in our study population. The *BRCA1 *UV carriers, but not the *BRCA2 *UV carriers, who were classified as high risk using each classification method were more similar to the deleterious mutation carriers with respect to family history than those carriers classified as low risk. For example, the odds ratio of having a first-degree family history for the high-risk women classified using Polyphen was 3.39 (95% confidence interval = 1.16 to 9.94) compared with normal/polymorphic *BRCA1 *carriers. The corresponding odds ratio of low-risk women was 1.53 (95% confidence interval = 1.07 to 2.18). The odds ratio for high-risk women defined by allele frequency was 2.00 (95% confidence interval = 1.14 to 3.51), and that of low-risk women was 1.30 (95% confidence interval = 0.87 to 1.93).

**Conclusion:**

The results suggest that the five classification methods yielded similar results. Polyphen was particularly better at isolating *BRCA1 *UV carriers likely to have a family history of breast cancer or ovarian cancer, and may therefore help to classify *BRCA1 *UVs. Our study suggests that these methods may not be as successful in classifying *BRCA2 *UVs.

## Introduction

In the early 1990s the breast cancer susceptibility genes *BRCA1 *and *BRCA2 *were identified through linkage analyses [1-4]. *BRCA1*, located on chromosome 17q12-q21, consists of 24 exons encoding a protein of 1,863 amino acids and is involved in DNA repair [5,6], in transcription [7,8], and in the cell cycle checkpoint in DNA damage response [9-11]. *BRCA2*, located on chromosome 13q12-q13, consists of 27 exons encoding a protein of 3,418 amino acids and is also involved in DNA repair [12-15], but its role in transcription and the cell cycle checkpoint is less clear [16].

Since the discovery of the *BRCA1 *and *BRCA2 *genes, a total of 1,643 and 1,856 distinct variants have been reported in the Breast Cancer Information Core (BIC) Database for *BRCA1 *and *BRCA2 *as of April 2007 [17]. Among these variants, frameshift mutations, nonsense mutations, splice variants and a few well-documented missense mutations are considered deleterious [18], while synonymous variants have been considered benign or polymorphic. A large number of missense or intronic variants of *BRCA1 *or *BRCA2 *remain of unknown significance. The proportion of breast cancer patients who carry these unclassified variants (UVs) is about 9% [19]. Given that only 2% to 3% of breast cancer patients have deleterious mutations in *BRCA1 *or *BRCA2 *[20], understanding the clinical significance of this relatively large number of UVs is of great importance.

Functional studies can provide direct insight into whether the UV has biological consequences, but few of these studies have been performed [21,22]. Other approaches have been applied to classify the significance of UVs, including comparisons of allele frequencies [18], algorithms such as Polyphen (see Materials and methods) [23], examination of sequence conservation across species [24-26], and characterization of the physicochemical nature of the amino acid substitutions (Grantham matrix scores) [26,27]. A combination approach of the sequence conservation and Grantham matrix score methods was applied to classify a large number of UVs [26]. No systematic evaluation, however, has been conducted to determine whether patients who carry the variants classified as high risk using these methods have similar characteristics as patients with known deleterious *BRCA1*/*BRCA2 *mutations, which would suggest that these high-risk UVs are deleterious.

Breast cancer patients with a known deleterious mutation in *BRCA1*/*BRCA2 *are more likely to have a family history of breast cancer or ovarian cancer [28] and an earlier age of diagnosis than noncarrier patients [18,29]. In addition, *BRCA1 *deleterious mutation carriers are more likely to have estrogen receptor (ER)-negative and progesterone receptor (PR)-negative tumors than women without such mutations [29]. In the current analyses, we classified *BRCA1*/*BRCA2 *UVs using the four methods listed above and a combination of the Grantham matrix scores and sequence conservation. We then evaluated the validity and usefulness of each method by comparing the risk categories of UV carriers with respect to these three well-defined characteristics of *BRCA1*/*BRCA2 *deleterious mutation carriers.

## Materials and methods

### Subjects

The data collection methods for this study have been described previously [30]. In brief, female patients diagnosed with histologically confirmed first primary invasive breast cancer were identified through the Los Angeles County Cancer Surveillance Program, a population-based Surveillance, Epidemiology and End Results registry supported by the State of California and the National Cancer Institute. Eligible cases were US born and English speaking, white (including Hispanic) or African-American, aged 20 to 49 years at diagnosis, and Los Angeles County residents at diagnosis. A total of 2,882 eligible cases were identified (2,534 whites and 348 African-Americans) between February 1998 and May 2003. Recruitment of African-Americans began after the initiation of the study with eligible African-American cases diagnosed from January 2000 to May 2003.

Among the 2,882 potentially eligible cases, 1,794 (62%) were interviewed (1,585 white, 209 African-American). Reasons for nonparticipation were patient refusal (*n *= 428), no longer a resident of Los Angeles County (*n *= 37), not located (*n *= 88), death (*n *= 38), serious illness or disability (*n *= 18), physician refusal (*n *= 50), or inability to schedule the interview within 18 months of diagnosis (*n *= 429). The study was approved by the Institutional Review Board of the University of Southern California. All participants provided written informed consent.

### Data and blood specimen collection

An inperson interview was completed using a modified version of the structured questionnaire used in the Women's Contraceptive and Reproductive Experiences Study [31]. The questionnaire included detailed information on demographic characteristics, family history of breast cancer or ovarian cancer, ethnic origin, and environmental factors such as oral contraceptive use, reproductive history, alcohol use, smoking history, and radiation exposure. We obtained information up to the date of breast cancer diagnosis. Blood specimens were collected from 1,519 participants (85%) and were transported to the Norris Cancer Center Genetics Core Laboratory in Styrofoam containers on frozen ice packs. For the first 50 samples the buffy coat was immediately extracted and stored, and for the remaining samples we stored whole blood.

### Sequencing of *BRCA1 *and *BRCA2 *genes

All *BRCA1 *and *BRCA2 *exons (except *BRCA1 *exons 1 and 4 and *BRCA2 *exon 1) as well as all exon–intron boundaries were sequenced. Exon 1 was not sequenced for either gene because it is located upstream of the translation start site in both genes. *BRCA1 *exon 4 was not sequenced because it is not found in the normal *BRCA1 *mRNA transcript.

DNA extraction, amplification and sequencing were carried out in the USC Genomics Core Laboratory using a protocol similar to that previously described [32]. The detailed procedures are described in the supplemental methods (see Additional File 1). We sequenced *BRCA1*/*BRCA2 *genes for 1,469 out of 1,519 blood specimens. We were unable to sequence the remaining 50 specimens due to insufficient DNA.

Thirty-three randomly selected, blinded samples were resequenced for quality control purposes. The discordance rate was 0.19%: 16 discordant sequencing results out of the total 8,646 variant sites sequenced (262 variant sites for each of the 33 samples). In addition, 166 subjects who had noninformative sequencing results on one or more variant sites were resequenced or genotyped using the TaqMan assay (for BRCA2 I2490T, N372H, and N991D) as previously described [33].

### Epidemiologic and histologic variables

Age at diagnosis was categorized as <35 years, 35 to 39 years, 40 to 44 years, and 45 to 49 years. We classified women based on their family history of breast cancer or ovarian cancer as follows: one or more breast cancer or ovarian cancer patients among their first-degree relatives (mother and full sisters); no first-degree family history of breast cancer or ovarian cancer but one or more breast cancer or ovarian cancer patients among their second-degree relatives (mother's or father's full sisters, and grandmothers); no first-degree or second-degree relatives diagnosed with breast cancer or ovarian cancer; and an unknown first-degree family history. We considered unknown second-degree family history as no family history.

The ER and PR status of the breast cancer was obtained by abstracting pathology reports collected by the Los Angeles County Cancer Surveillance Program. Among the 1,469 subjects, ER/PR information was available for 1,216 patients (83%). For the ER/PR analyses, we excluded 63 patients who had borderline ER/PR status and 101 patients whose ER/PR status was +/- or -/+, leaving 1,052 patients with a +/+ or -/- receptor status.

### Classification of *BRCA1*/*BRCA2 *mutation status

We classified each identified *BRCA1*/*BRCA2 *variant according to its predicted functional and biological significance as follows: definitely disease-causing variants (DDCVs), including frameshift mutations, nonsense mutations, splice variants that were previously reported to affect splicing or were located at the exon/intron boundary, and missense variants that were previously shown to be deleterious; UVs, including inframe deletion/insertions, intronic variants that might affect splicing by creating a splice donor/acceptor site, variants next to the exon/intron boundary, and most missense variants; and benign polymorphic variants, including synonymous variants, intronic variants that are unlikely to affect splicing, and a few missense mutations that were reported to be benign. (See Additional File 2 for a list of all variants identified in this study, with their classification and the reasons and references for such classification.)

### Further classification of *BRCA1 *and *BRCA2 *unclassified variants

We further classified *BRCA1*/*BRCA2 *UVs using the following methods.

#### Classification based on allele frequency

We divided the UVs into high-frequency unclassified variants (HFUVs) and low-frequency unclassified variants (LFUVs) depending on the minor allele frequency (≥ 1% versus <1%) in each ethnic group (142 African-Americans, 222 Hispanic whites, 1,105 non-Hispanic whites). If the minor allele frequency is ≥ 1% in one or more ethnic groups, the UV was categorized as a HFUV. This categorization was based on the assumption that variants with high frequency would be less likely to be disease causing compared with variants of very low frequency.

#### Polyphen-based classification

Polyphen is an algorithm that classifies the functional effect of each missense variant into three categories (probably damaging, possibly damaging, and benign) [34]. This classification is based on the chemical characteristics of the substitution site (for example, disulfide bond, transmembrane region), the alignment of homologous sequences, and protein three-dimensional structures [23]. UVs other than missense variants are not classified by Polyphen. The Polyphen classification in this report is based on access to the algorithm in March 2007.

#### Classification based on sequence conservation across mammalian species

A variant that occurs at a site with high-degree conservation is considered more likely to be deleterious than a variant occurring at a site with low-degree conservation [35]. We selected only mammals for cross-species comparisons of the *BRCA1*/*BRCA2 *sequences, since the function of these two proteins in mammals could be different from that in other animals. We selected all mammalian species whose *BRCA1*/*BRCA2 *sequences were reported in the National Center for Biotechnology Information gene database or whose complete coding sequences were reported in the National Center for Biotechnology Information nucleotide sequence database. Ten species for *BRCA1 *and five species for *BRCA2 *met these criteria (see Additional File 2). Sequence alignment was performed using the Clustal W method [36] and the MegAlign software (DNASTAR, Inc., Madison, WI, USA).

We classified *BRCA1*/*BRCA2 *missense variants into three categories (high conservation, moderate conservation, and low conservation) depending on the number of the species that had a different amino acid from that of the human at the site of variation. For each UV in *BRCA1 *we considered differences in zero or one species out of the 10 examined to represent high conservation, differences in two or three species to represent moderate conservation, and differences in four or more species to represent low conservation. For *BRCA2 *we compared sequences of five species: no difference in all five species was considered high conservation, one or two differences were considered moderate conservation, and three or more differences were considered low conservation.

#### Classification based on the Grantham matrix score

The Grantham matrix score (GMS) is a composite measure of the degree of amino acid substitution, taking into account the side-chain composition, polarity, and molecular volume of the two amino acids [27]. We dichotomized the GMS at 60, a criterion previously used to define neutral missense variants [26].

#### Integration of sequence conservation and the Grantham matrix score

We adopted a previously reported classification scheme integrating the sequence conservation and the GMS [26]. Briefly, if the variant was located at a fully conserved site or led to a nonconservative substitution at a conserved site, it was considered deleterious. If the variant amino acid is observed in other species or led to conservative substitution, it was considered neutral. See Additional File 1 for further details.

### Classification of women who carry unclassified variants in *BRCA1*/*BRCA2*

**Figure 1:**
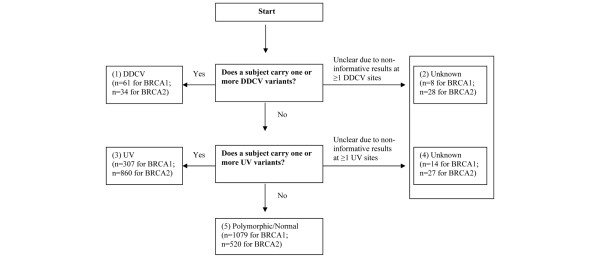
**Illustration of the classification scheme of *BRCA1*/*BRCA2 *variants**. DDCV, definitely disease-causing variant; UV, unclassified variant.

Each subject was categorized hierarchically based on their *BRCA1 *and *BRCA2 *mutation status (Figure 1). This means that anyone successfully classified by the first criterion would not be further classified by the criteria that followed. This hierarchical classification leads to mutually exclusive categories (DDCV carriers, UV carriers, normal/polymorphic carriers, and patients with unknown mutation status) as follows. First, a patient was classified as a DDCV carrier if she had one or more of the DDCV(s). Second, if the patient did not belong to the DDCV group and had a noninformative result at any of the identified DDCV sites, she was classified as unknown. Third, if the patient did not belong to these first two categories and carried one or more of the UVs, she was classified as a UV carrier. Fourth, if the patient did not belong to the first three categories and any of the sequencing results at the identified UV sites was noninformative for the subject, she was classified as unknown. Finally, if the patient did not belong to any of the preceding categories, she was classified as a polymorphic or normal genotype carrier.

UV carriers were further classified hierarchically into mutually exclusive categories of high risk, moderate risk, low risk, and unknown risk according to the various UV classifications. For example, when applying the allele frequency method, a UV carrier was classified as high risk if the subject carried one or more of the LFUVs, as unknown risk if any of the sequencing results at the LFUV site was noninformative for the subject, as low risk if the subject carried one or more of the HFUVs, and as unknown risk if any of the sequencing results at the HFUV site was noninformative for the subject. Classification using other methods such as Polyphen, the GMS, or sequence conservation followed the same hierarchical logic.

Six *BRCA1 *UV carriers and six *BRCA2 *UV carriers with a possible splice variant or in-frame deletion were categorized only by allele frequency since Polyphen, the GMS, and the integrated GMS/sequence conservation methods are not applicable to these splice variants and in-frame deletions. These women were therefore excluded from the analyses using Polyphen, the GMS, sequence conservation, and the integrated GMS/sequence conservation methods.

### Statistical analyses

We compared the UV classification methods of allele frequency, Polyphen, sequence conservation, and the GMS by examining the pairwise joint distribution of *BRCA1*/*BRCA2 *UVs as classified using each method. Tests for a linear trend in the GMS across the three UV categories classified using Polyphen and the sequence conservation method were conducted in linear regression models. The mean GMS across two UV categories using allele frequency was compared by *t *test. We assessed whether UV classifications using allele frequency, Polyphen and the sequence conservation methods are correlated using an exact Mantel–Haenszel chi-square test.

We performed case–case analyses to examine the association between *BRCA1 *or *BRCA2 *carrier status categorized using each method (exposure variable) and outcome variables (clinical and disease characteristics). Case–case analyses were conducted using polychotomous logistic regression when the outcome variable was family history of breast cancer or ovarian cancer. The association with the ER/PR status was analyzed using logistic regression. We used linear regression where the outcome variable was age at diagnosis of breast cancer. When examining *BRCA1*, results were adjusted for the *BRCA2 *mutation status (DDCV, non-DDCV, unknown), and *vice versa*.

All reported *P *values are two-sided. The SAS 9.1 package was used for all analyses (SAS Institute, Cary, NC, USA).

## Results

A total of 105 distinct *BRCA1 *variants (including 32 DDCVs) and 157 distinct *BRCA2 *variants (including 27 DDCVs) were identified in the 1,469 breast cancer patients (see Additional File 3). Among these distinct variants, 22 *BRCA1 *variants and 30 *BRCA2 *variants had not been reported in the BIC as of April 2007.

### Correlated classifications using various approaches

Classification using the Polyphen algorithm appeared to be correlated both with the GMS and the conservation method: *BRCA1*/*BRCA2 *missense variants classified as high risk (probably damaging) using Polyphen had a higher mean GMS than those classified as low risk (benign missense variants) (Table 1). *BRCA1*/*BRCA2 *missense variants classified as benign missense variants using Polyphen were generally located at sites with low degree of sequence conservation, while probably damaging missense variants tended to be located in highly conserved regions (Table 2). The GMS, however, was not strongly correlated with level of conservation across species (Table 1). Given the small number of HFUVs of *BRCA1*/*BRCA2*, the classification using the allele frequency method seemed to be associated with the classifications using other methods, although not all of these analyses achieved statistical significance.

**Table 1:** Mean Grantham matrix score of *BRCA1*/*BRCA2 *variants (unclassified variants) according to classification using allele frequency, Polyphen, and sequence conservation

Gene	Classification method	Level	Number of variants	Grantham matrix score			
				
				Mean	Standard deviation	Minimum	Maximum
*BRCA1*	Allele frequency	Low risk (HFUV)	5	38.2	20.7	10	56
		High risk (LFUV)	39	69.7	45.0	10	194
		*P *value (*t *test)		0.13			
	Polyphen	Benign missense	28	55.0	42.3	10	180
		Possible damaging	9	65.3	29.4	10	101
		Probably damaging	7	111.9	40.5	71	194
		*P *for trend^a^		0.002			
	Sequence conservation	Low	14	53.6	44.7	10	180
		Moderate	14	70.9	43.5	10	154
		High	16	73.0	44.2	10	194
		*P *for trend^a^		0.24			
*BRCA2*	Allele frequency	Low risk (HFUV)	18	67.7	39.7	5	149
		High risk (LFUV)	77	87.0	52.9	10	205
		*P *value (*t *test)		0.15			
	Polyphen	Benign missense	44	58.1	41.3	5	194
		Possible-damaging	25	91.4	41.7	21	180
		Probably-damaging	26	118.3	52.3	27	205
		*P *for trend^a^		<0.001			
	Sequence conservation	Low	33	71.5	49.4	10	194
		Moderate	29	92.1	55.6	5	205
		High	33	87.5	47.8	21	194
		*P *for trend^a^		0.20			

LFUV, low-frequency unclassified variant; HFUV, high-frequency unclassified variant. ^a^Based on the *F *test in a linear regression model.

**Table 2:** Joint distribution of *BRCA1*/*BRCA2 *variants (unclassified variants) according to classification using allele frequency, Polyphen, and sequence conservation

Gene	Classification method	Level	Conservation			Frequency	
				
			Low	Moderate	High	HFUV	LFUV
*BRCA1*	Frequency	Low risk (HFUV)	3	1	1		
		High risk (LFUV)	11	13	15		
		*P *value^a^			0.26		
	Polyphen	Benign missense	12	10	6	4	24
		Possible damaging	2	3	4	1	8
		Probably damaging	0	1	6	0	7
		*P *value^a^			0.002		0.39
*BRCA2*	Frequency	Low risk (HFUV)	10	6	2		
		High risk (LFUV)	23	23	31		
		*P *value^a^			0.018		
	Polyphen	Benign missense	26	11	7	11	33
		Possible damaging	5	10	10	5	20
		Probably damaging	2	8	16	2	24
		*P *value^a^			<0.001		0.089

LFUV, low-frequency unclassified variant; HFUV, high-frequency unclassified variant. ^a^Based on the exact Mantel–Haenszel chi-square test.

### Classification of case patients with regard to *BRCA1 *or *BRCA2 *status

Among the 1,469 case patients in this study, 61 women carried a *BRCA1 *DDCV and 34 women carried a *BRCA2 *DDCV. Among the remaining women, 307 women and 860 women were UV carriers in *BRCA1 *and in *BRCA2*, respectively.

### Classification of *BRCA1*/*BRCA2 *status in relation to epidemiologic and histologic outcome variables

#### Family history of breast cancer or ovarian cancer

The *BRCA1 *DDCV carriers were substantially more likely to have a first-degree family history of breast cancer or ovarian cancer than the normal/polymorphic *BRCA1 *carriers (odds ratio = 11.3; Table 3) after adjusting for the *BRCA2 *mutation status. The UV carriers were also significantly, although to a smaller extent, more likely to have a first-degree family history than normal/polymorphic *BRCA1 *carriers (odds ratio = 1.54). The high-risk UV carriers were, in general, significantly more likely to have a first-degree family history of breast cancer or ovarian cancer than normal/polymorphic women, whereas the low-risk UV carriers were not. For example, the high-risk UV carriers identified using the allele frequency (LFUV) or Polyphen (probably damaging) methods were more likely to have a first-degree family history (odds ratio = 2.00 and 3.39, respectively) than normal/polymorphic *BRCA1 *carriers.

**Table 3:** Association between family history of breast cancer or ovarian cancer and *BRCA1 *or *BRCA2 *status of the breast cancer patients

Mutation/unclassified variant status	None	First degree			Second degree		
			
	*n*^a^	*n*^a^	OR (95% CI)^b^	*P *value^c^	*n*^a^	OR (95% CI)^b^	*P *value^c^
*BRCA1*^d^							
Normal/polymorphism (reference)	600	166	1		282	1	
Definitely disease-causing variant	13	35	11.3 (5.73 to 22.5)	<0.001	11	1.89 (0.83 to 4.31)	0.13
Unclassified variant	156	67	1.54 (1.10 to 2.15)	0.012	75	1.02 (0.74 to 1.39)	0.90
Unclassified variant classification using							
Allele frequency							
High risk (LFUV)	39	21	2.00 (1.14 to 3.51)	0.016	22	1.20 (0.70 to 2.06)	0.52
Low risk (HFUV)	115	42	1.30 (0.87 to 1.93)	0.20	52	0.96 (0.67 to 1.37)	0.82
Polyphen^e^							
Probably damaging missense	7	7	3.39 (1.16 to 9.94)	0.026	6	1.80 (0.60 to 5.40)	0.30
Possibly damaging missense	9	1	0.44 (0.06 to 3.54)	0.44	5	1.19 (0.40 to 3.60)	0.75
Benign missense	133	57	1.53 (1.07 to 2.18)	0.021	61	0.97 (0.70 to 1.36)	0.87
Sequence conservation^e^							
High or moderate^f^	50	23	1.68 (0.99 to 2.85)	0.053	32	1.35 (0.85 to 2.16)	0.20
High	6	3	1.88 (0.46 to 7.63)	0.38	8	2.85 (0.98 to 8.30)	0.055
Moderate	44	20	1.66 (0.95 to 2.91)	0.076	23	1.10 (0.65 to 1.86)	0.72
Low	99	40	1.43 (0.95 to 2.16)	0.085	41	0.88 (0.60 to 1.31)	0.53
Grantham matrix score^e^							
High (>60)	15	10	2.38 (1.04 to 5.45)	0.039	10	1.42 (0.63 to 3.20)	0.40
Low (≤ 60)	134	55	1.46 (1.02 to 2.10)	0.039	63	1.00 (0.72 to 1.39)	0.98
Grantham matrix score/sequence conservation^e^							
Deleterious	4	2	1.74 (0.32 to 9.64)	0.52	3	1.63 (0.36 to 7.33)	0.53
Intermediate (unclassified)	46	22	1.75 (1.02 to 3.01)	0.043	25	1.15 (0.69 to 1.91)	0.60
Neutral	99	40	1.44 (0.95 to 2.16)	0.083	44	0.95 (0.65 to 1.39)	0.78
*BRCA2*^g^							
Normal/polymorphism (reference)	279	87	1		137	1	
Definitely disease-causing variant	11	13	3.69 (1.57 to 8.68)	0.003	10	1.83 (0.76 to 4.42)	0.18
Unclassified variant	462	162	1.07 (0.79 to 1.46)	0.66	213	0.93 (0.72 to 1.21)	0.59
Unclassified variant classification using							
Allele frequency							
High risk (LFUV)	71	18	0.81 (0.45 to 1.45)	0.48	40	1.15 (0.74 to 1.78)	0.54
Low risk (HFUV)	385	138	1.09 (0.79 to 1.50)	0.59	168	0.88 (0.67 to 1.16)	0.36
Polyphen^e^							
Probably damaging missense	32	7	0.73 (0.31 to 1.74)	0.48	17	1.09 (0.58 to 2.03)	0.79
Possibly damaging missense	108	28	0.79 (0.48 to 1.29)	0.34	42	0.78 (0.52 to 1.18)	0.24
Benign missense	310	118	1.15 (0.83 to 1.60)	0.41	146	0.95 (0.72 to 1.26)	0.73
Sequence conservation^e^							
High or moderate	157	42	0.80 (0.52 to 1.23)	0.31	61	0.78 (0.55 to 1.12)	0.18
High	96	30	0.92 (0.56 to 1.51)	0.75	41	0.86 (0.57 to 1.31)	0.48
Moderate	61	12	0.61 (0.31 to 1.21)	0.15	20	0.66 (0.38 to 1.14)	0.13
Low	292	113	1.18 (0.84 to 1.65)	0.33	142	0.98 (0.74 to 1.31)	0.91
Grantham matrix score^e^							
High (>60)	450	155	1.05 (0.77 to 1.43)	0.76	205	0.92 (0.71 to 1.20)	0.53
Low (≤ 60)	9	3	0.98 (0.25 to 3.85)	0.98	5	1.11 (0.37 to 3.39)	0.85
Grantham matrix score/sequence conservation^e^							
Deleterious	124	34	0.83 (0.52 to 1.31)	0.42	51	0.83 (0.56 to 1.22)	0.34
Intermediate (unclassified)	66	14	0.67 (0.35 to 1.27)	0.22	28	0.87 (0.53 to 1.41)	0.56
Neutral	259	107	1.24 (0.89 to 1.75)	0.21	124	0.97 (0.72 to 1.30)	0.82

OR, odds ratio; CI, confidence interval; LFUV, low-frequency unclassified variants; HFUV, high-frequency unclassified variants. ^a^Twenty-two and 55 women with unknown *BRCA1 *and *BRCA2 *status were included as a separate category for corresponding analyses. Numbers do not add up when further classifying unclassified variants (UVs) using the classification methods since the UV carriers who had missing values for higher-risk UV categories could not be categorized into high-risk or low-risk groups and thus were added to the unknown group. ^b^Family history of breast cancer or ovarian cancer is the outcome (no family history (reference), first degree, and second degree). Family history unknown cases were deleted from the analysis. All analyses were adjusted for age at diagnosis (<35 years, 35 to <40 years, 40 to <45 years, 45+ years). ^c^Based on the chi-square test. ^d^When analyzing *BRCA1*, the model was further adjusted for the *BRCA2 *mutation status (definitely disease-causing variant, non-definitely disease-causing variant, unknown). ^e^Splice/inframe deletion carriers of *BRCA1 *were excluded when analyzing *BRCA1*. Splice/inframe deletion carriers of *BRCA2 *were excluded when analyzing *BRCA2*. ^f^Numbers do not add up due to one additional subject classified after combining the high and moderate groups who was in the unknown *BRCA1 *status before combining. ^g^When analyzing *BRCA2*, the model was further adjusted for *BRCA1 *mutation status (definitely disease-causing variant, non-definitely disease-causing variant, unknown).

A similar trend was observed using the sequence conservation or the GMS method, although differences between the categories of UV carriers were smaller. The integrated method of the GMS/sequence conservation classified only nine subjects as high risk, and their odds ratio was not different from that of the women who remained unclassified.

The *BRCA2 *DDCV carriers were also at a higher risk of having a first-degree family history of breast cancer or ovarian cancer compared with the normal/polymorphic *BRCA2 *carriers (odds ratio = 3.69) after adjusting for *BRCA1 *mutation status. The association was weaker than that of *BRCA1 *DDCV carriers. Regardless of the classification method, the high-risk UV carriers were not statistically significantly different from the normal/polymorphic *BRCA2 *carriers with regard to family history (Table 3).

#### Age at diagnosis and estrogen receptor/progesterone receptor status

As expected, compared with the carriers of normal/polymorphic *BRCA1*, the *BRCA1 *DDCV carriers had a much earlier age at diagnosis (by 4.1 years; *P *< 0.001) and more ER/PR-negative tumors (odds ratio = 7.24, 95% confidence interval = 3.56 to 14.7). Case patients with high-risk UVs, however, did not have such characteristics regardless of the method of UV classification. The *BRCA2 *DDCV or UV status was not associated with early age at diagnosis or with ER/PR negativity (data not shown).

### Comparisons of the classifications using the methods in this study and the Breast Cancer Information Core

The recent update of the BIC includes the assessment of the clinical importance of each variant. This assessment is based on several criteria, including epidemiological, segregation, and co-occurrence data. Among the UVs in this study, one *BRCA1 *UV (IVS5-11T > G) was classified as clinically important whereas three *BRCA1 *UVs and 19 *BRCA2 *UVs were classified as clinically nonimportant. IVS5-11T > G was classified as a high-risk UV using allele frequency (LFUV). Since this variant is not a missense variant, other methods were not applicable. Table 4 shows how each UV that was considered nonimportant in the BIC was classified by the five UV classification methods. The allele frequency and the GMS method classified a large number of variants as high risk that were considered nonimportant by the BIC, particularly for *BRCA2*. In contrast, Polyphen and the conservation methods classified few such variants as high risk.

**Table 4:** Classification of *BRCA1*/*BRCA2 *variants (unclassified variants) that were considered clinically not important in the Breast Cancer Information Core database

Unclassified variant classification method	Number of *BRCA1 *variants	Number of *BRCA2 *variants
Allele frequency		
High risk (LFUV)	1 (1200H)	15 (S326R, S384F, D596H, T598A, S976I, C1290Y, D1420Y, G1529R, H2116R, T2515I, A2717S, V2728I, S2835P, E2856A, T3013I)
Low risk (HFUV)	2 (I379M, D693N)	6 (N372H, N289H, L929S, N987I, N991D, T1414M)
Polyphen		
High risk (probable)	0	3 (N987I, C1290Y, G1529R, H2116R)
Medium risk (possible)	1 (I379M)	8 (N289H, N372H, S384F, D596H, S976I, D1420Y, T2515I, E2856A)
Low risk (benign)	2 (D693N, 1200H)	9 (S326R, T598A, L929S, N991D, T1414M, A2717S, V2728I, S2835P, T3013I)
Sequence conservation		
High risk (high conservation)	1 (I379M)	4 (N289H, D596H, G1529R, E2856A)
Medium risk (moderate conservation)	1 (Q1200H)	10 (S326R, T598A, V2728I, S384F, S976I, N987I, C1290Y, D1420Y, H2116R, T2515I)
Low risk (low conservation)	1 (D693N)	7 (N372H, L929S, N991D, T1414M, A2717S, S2835P, T3013I)
Grantham matrix score		
High risk (>60)	1 (Q1200H)	17 (N289H, S326R, N372H, S384F, D596H, L929S, S976I; C1290Y, T1414M, D1420Y, G1529R, T2515I, A2717S, S2835P, N987I, E2856A, T3013I)
Low risk (≤ 60)	2 (I379M, D693N)	4 (T598A, N991D, H2116R, V2728I)
Grantham matrix score/sequence conservation		
High risk (deleterious)	0	7 (N289H, D596H, L929S, N987I, 1420Y, G1529R, E2856A)
Medium risk (unclassified)	2 (I379M, 1200H)	7 (S326R, S384F, S976I, H2116R, T2515I, A2717S, T3013I)
Low risk (neutral)	1 (D693N)	7 (N372H, T598A, N991D, C1290Y, T1414M, V2728I, S2835P)

LFUV, low-frequency unclassified variants; HFUV, high-frequency unclassified variants.

## Discussion

In the present study of young breast cancer patients, we identified numerous variants in *BRCA1*/*BRCA2 *by direct sequencing, including 22 *BRCA1 *and 30 *BRCA2 *new variants that have not been reported in the BIC as of April 2007. We applied various methods to classify 44 *BRCA1 *UVs and 95 *BRCA2 *UVs. To our knowledge, our study is the first to attempt to classify a large number of *BRCA1*/*BRCA2 *UVs identified in population-based breast cancer patients and to correlate these variants with outcome variables.

We found that classifications of *BRCA1*/*BRCA2 *UVs using the various classification methods in general agree with each other (Table 1 and Table 2). In particular, Polyphen seemed to be correlated with the GMS and with sequence conservation, which is expected given the composite nature of this algorithm. This intercorrelation supports the reliability of the classification methods.

In general, the *BRCA1 *UV carriers classified as high risk were at increased risk of having a family history of breast cancer or ovarian cancer. Family history has been considered a powerful tool in classifying UVs [37], and having a first-degree relative with breast cancer increases the breast cancer risk about twofold [38]. The odds ratio for the high-risk UV group was highest when using Polyphen, suggesting that the algorithm is better for the purpose of describing high-risk variants when using family history as a measure of true risk. We cannot exclude, however, the possibility that more stringent cutoff points to define the high-risk group using other methods (that is, high-degree conservation defined as no cross-species variation; or high GMS defined as >100) might increase the odds ratio estimates of the high-risk group. In this study, we did not have sufficient numbers of UV carriers to investigate this possibility.

Considering that the high-risk *BRCA1 *UV carriers classified using all of the classification methods were at a higher risk of having a family cancer history (either statistically significantly or nonsignificantly), we expected to observe similar trends using age of diagnosis or the ER/PR status as the outcome variables. This observation, however, did not occur. The narrow age range of our study subjects, all of whom were under age 50 at diagnosis, could have limited the study power. For analyses of the ER/PR status, our exclusion of about 30% of women because of missing, borderline, or mixed (-/+ or +/-) ER/PR status may have limited the statistical power. Alternatively, it is possible that only truncating mutations (resulting in a complete loss of *BRCA1 *functions), but not missense variants (retaining part of its ability; for example, the ability to interact with certain proteins), of *BRCA1 *lead to the high density of ER/PR-negative tumors.

For *BRCA2*, it is unclear why none of the classification methods identified high-risk UV carriers when family history was used as the measure of true risk. One explanation could be the fact that *BRCA2 *DDCV carriers themselves did not have such a high odds ratio as seen for *BRCA1 *DDCV carriers. The *BRCA2 *DDCV carrier status was also not associated with age at diagnosis in this study, again possibly because all of our subjects were younger than 50 years and the age at diagnosis for *BRCA2 *DDCV carriers is not as early as for *BRCA1 *DDCV carriers [29]. In our study, the median ages were 40 and 45 years for *BRCA1 *and *BRCA2 *DDCV carriers, respectively.

Homozygous deleterious mutations in *BRCA1*/*BRCA2 *are lethal [39-42]. In the present study, all of the low-risk UVs classified using the allele frequency method (except those that were common only in African-Americans) were observed as homozygous and therefore should be benign. Consistent with this, all our low-risk UVs (HFUVs) that have been classified by the BIC were assessed as clinically nonimportant. On the contrary, quite a few variants classified by the BIC as nonimportant are rare variants, and are therefore classified as high-risk UVs (LFUVs) in our study. If a variant has arisen very recently, its population frequency will be low even though the variant is not clinically important [43]. The allele-frequency method may therefore be better for the purpose of describing low-risk UVs than high-risk UVs.

The GMS is a pairwise comparison of the two substituted amino acids, and it has been argued that a multiple comparison – that is, a comparison of the substituted amino acids taking into account the natural variation of the substituted site across species – would provide better information [44,45]. One method of achieving such a multiple comparison is to use the integrated method of Abkevich and colleagues [26]. In our study, however, this method was not an improvement over the individual application of the two methods.

The Polyphen algorithm compares homologous sequences for conservation and examines the structural and physicochemical aspects of the substitution. We found that the high-risk UV carriers identified using Polyphen had the highest odds ratio of first-degree family history among those identified using all other methods. We also found that the number of clinically nonimportant variants that were classified as high risk or medium risk was smallest when using Polyphen. The Polyphen algorithm has been reported to have the smallest false-positive rate among the various online algorithms, including SIFT [35]. Polyphen has previously not been applied for *BRCA1*/*BRCA2 *whereas SIFT has been adopted for *BRCA1 *[24,25]. Our results suggest that Polyphen might be useful to identify high-risk UVs, especially when the UV has never been reported and/or clinical information is not available.

Efforts to classify UVs are accumulating: several groups have used simple combinations of sequence conservation and the severity of amino acid substitutions [24-26]. Whether the classification is clinically valid, however, has not been systematically examined [26]. Other studies have used extensive multifactorial models, most of them focusing on a few *BRCA1 *UVs. These models incorporate several approaches used in this study as well as clinical characteristics [46], co-occurrence with deleterious mutations [19,46], and histopathological information [19]. While clinical and co-occurrence information has provided strong evidence to classify UVs [37,46], however, such information is not always available, especially for UVs that have not been reported before. Further, it has been suggested that these "ideal" criteria cannot classify the majority of the UVs [37]. The classification methods used in the present study may serve as "readily available" additional information to classify Uvs.

## Conclusion

The present study suggests that the application of different methodologies such as allele frequency, Polyphen, the GMS, and sequence conservation may be useful for evaluating UVs, especially when little functional or clinical data are available. While we found high correlations between these classification methods, our study suggests that each method has different levels of false-positives and false-negatives. The Polyphen algorithm appeared more appropriate in identifying high-risk variants whereas the allele frequency may be useful in classifying high-frequency variants as nonimportant. Although our study does not directly address the question of whether each specific UV is associated with the risk of breast cancer, our results suggest that these methods could be helpful in understanding the significance of a UV especially when other clinical or genetic information is not available. Further, the application of these methods may help to prioritize UVs for further functional or familial study.

## Abbreviations

BIC = Breast Cancer Information Core; DDCV = definitely disease-causing variant; ER = estrogen receptor; GMS = Grantham matrix score; HFUV = high-frequency unclassified variant; LFUV = low-frequency unclassified variant; PR = progesterone receptor; UV = unclassified variant.

## Competing interests

The authors declare that they have no competing interests.

## Authors' contributions

EL cleaned the data, classified *BRCA1*/*BRCA2 *unclassified variants, performed the data analysis and drafted the manuscript. RM-C participated in classification of the *BRCA1*/*BRCA2 *variants and revision of the manuscript. HM and ZC cleaned the data, and participated in classification of the *BRCA1*/*BRCA2 *variants and revision of the manuscript. DVDB sequenced the *BRCA1*/*BRCA2 *genes, classified *BRCA1*/*BRCA2 *variants, and participated in revision of the manuscript. LB participated in the design of the study and data collection, and revised the manuscript. BEH participated in the design and conception of the study, and supported the laboratory work. GU designed the study, supervised the data collection, participated in *BRCA1*/*BRCA2 *classification, supervised the data analysis, and revised the manuscript. All authors read and approved the final manuscript.

## Supplementary Material

Additional file 1 Word file containing the detailed sequencing procedures and the classification approach integrating sequence conservation and the GMS. Click here for file

Additional file 2 Word file containing a table listing the protein sequences used for cross-species comparison of *BRCA1 *and *BRCA2*. Click here for file

Additional file 3 Word file containing a table listing all of the *BRCA1*/*BRCA2 *variants identified in this study along with our classification of each variant in comparison with the classification according to the BIC database. Click here for file
