# Supplementary material for: Evaluation of unclassified variants in the breast cancer susceptibility genes BRCA1 and BRCA2 using five methods: results from a population-based study of young breast cancer patients
Source: Breast Cancer Res. 2008 Feb 19;10(1):R19. doi: 10.1186/bcr1865 (PMC2374975; doi:10.1186/bcr1865)
Supplement: Additional file 1 — Word file containing the detailed sequencing procedures and the classification approach integrating sequence conservation and the GMS. [file bcr1865-S1.doc]

**Sequencing of BRCA1 and BRCA2 genes:** DNA extraction, amplification and sequencing was done in the USC Genomics core laboratory under the supervision of Dr. David Van Den Berg (D.V.D.B.) using a protocol similar to that previously described (1).

*DNA extraction and PCR amplification*: DNA extraction from 200 μl buffy coat was performed using a QIAamp 96 Blood kit (Qiagen Inc., Valencia, CA). All BRCA1 and BRCA2 exons, except BRCA1 exons 1 and 4 and BRCA2 exon 1, and each exon-intron boundaries were amplified by PCR using exon-specific primers (information on primers will be provided upon request). Exon 1 for BRCA1 and BRCA2 was not amplified because it is located upstream of translation start site, and BRCA1 exon 4 was not amplified because it is not found in normal BRCA1 mRNA trascript. Each PCR reaction contained ~30 ng of genomic DNA, 40 pmoles of forward and reverse primer, 100 μM dNTPs, 2 U Ampli*Taq* Gold DNA polymerase (Applied Biosystems, Foster City, CA), 1× Gold buffer A and MgCl_2_ in a total volume of 25 μl. The PCR mixture was denatured at 94°C for 1 min (except the initial denaturing for 12 min), annealed for 1 min at a temperature varying from 52°C to 58°C depending on the melting temperature of the exon-specific primer pairs, and extended at 72°C for 1 min (except the extension for the final cycle for 10min) for 35 cycles. Amplification of exon 11 was performed in a total volume of 100 μl using a GeneAmp XL PCR kit (Applied Biosystems Inc.). All PCR reactions were performed in 96-well plates using a Primus 96 cycler (MWG Biotech, High Point, NC). The amplified PCR products were purified using MultiScreen FB filter plates (Millipore Corporation, Billerica, MA), and eluted in 70-140 μl double-distilled H_2_O. The presence of the PCR product for each PCR reaction was measured by agarose-gel electrophoresis using 16 samples from each plate.

*Sequencing reactions*: We sequenced the purified PCR products with an ABI PRISM BigDye Terminator Cycle Sequencing Ready Reaction Kit (Applied Biosystems Inc.). The reactions were perfomed using five to 10 ng of the purified PCR products, 10.4 pmoles of forward or reverse primers (for bi-directional sequencing), Ready Reaction Premix and 1× reaction buffer in a total volume of 12 μl. Cycle sequencing reactions were performed in a Primus 96 cycler at 96°C for 10 s, annealing at 50°C for 5 s, and extension at 60°C for 4 min were performed for 25 cycles. Prior to capillary electrophoresis, unincorporated dye terminators were removed from the extension product using a DyeEx 96 Plate (Qiagen, Inc.) according to the manufacturer’s instructions. The purified extension products were denatured at 90°C for 2 min and placed on ice for 1 min. Sequencing was performed on an ABI PRISM 3700 or 3730xl DNA Analyzer (Applied Biosystems Inc.).

We processed the data collected from the ABI detection system using the Phred/Phrap software developed at the University of Washington (2-5). Sequence alignments for each exon read were viewed in the Consed viewer Software and sequence variations were annotated and recorded (DVDB). Negative controls were checked for the absence of analyzable sequence. If a sequence was present in the negative control, the position of where the negative control would have been if a plate flip occurred (NC-flip) was checked for analyzable sequence. If the NC-flip was negative, the plate was analyzed as a flip. If the NC-flip was positive, then the sequencing reactions were rerun. Sequence variation for the forward and reverse read were compared and any discordant findings were checked to determine which read was correct. Sequence variations were summarized for each plate using Microsoft Excel and merged into a single file after completion of each 96-well plate of samples.

**Further classification of BRCA1 and BRCA2 Unclassified Variants (UVs) using the integrated method of sequence conservation and GMS:** We adopted a previously-reported classification scheme integrating the sequence conservation and GMS (6). Briefly, if the variant was located at a fully conserved site, it was considered deleterious. In addition, if the variant involved non-conservative or severe substitution at a conserved site, that is (i) the maximum GMS between a pair of aligned sequences at the site of variation is not greater than 60 and (ii) GMS for human variant is greater than or equal to three times of the maximum GMS in the alignment, then it was considered deleterious. If the variant amino acid is observed in other species, it was considered neutral. In addition, if the variant involves conservative substitution relative to natural variation across species, that is (i) GMS for human variant is smaller than 61 and (ii) GMS for human variant is smaller than one third of the maximum GMS in the alignment, it was considered neutral.

1. McKean-Cowdin, R., Spencer Feigelson, H., Xia, L. Y., Pearce, C. L., Thomas, D. C., Stram, D. O., and Henderson, B. E. BRCA1 variants in a family study of African-American and Latina women. Hum Genet, *116:* 497-506, 2005.

2. Ewing, B. and Green, P. Base-calling of automated sequencer traces using phred. II. Error probabilities. Genome Res, *8:* 186-194, 1998.

3. Ewing, B., Hillier, L., Wendl, M. C., and Green, P. Base-calling of automated sequencer traces using phred. I. Accuracy assessment. Genome Res, *8:* 175-185, 1998.

4. Gordon, D., Abajian, C., and Green, P. Consed: a graphical tool for sequence finishing. Genome Res, *8:* 195-202, 1998.

5. Nickerson, D. A., Tobe, V. O., and Taylor, S. L. PolyPhred: automating the detection and genotyping of single nucleotide substitutions using fluorescence-based resequencing. Nucleic Acids Res, *25:* 2745-2751, 1997.

6. Abkevich, V., Zharkikh, A., Deffenbaugh, A. M., Frank, D., Chen, Y., Shattuck, D., Skolnick, M. H., Gutin, A., and Tavtigian, S. V. Analysis of missense variation in human BRCA1 in the context of interspecific sequence variation. J Med Genet, *41:* 492-507, 2004.
