# Supplementary material for: Evaluation of unclassified variants in the breast cancer susceptibility genes BRCA1 and BRCA2 using five methods: results from a population-based study of young breast cancer patients
Source: Breast Cancer Res. 2008 Feb 19;10(1):R19. doi: 10.1186/bcr1865 (PMC2374975; doi:10.1186/bcr1865)
Supplement: Additional file 2 — Word file containing a table listing the protein sequences used for cross-species comparison of BRCA1 and BRCA2. [file bcr1865-S2.doc]

Supplemental Table 1. Sequences used for cross-species comparison of BRCA1 and BRCA2.

| Species | mRNA | protein | length |
| --- | --- | --- | --- |
| BRCA1 ^§^ |  |  |  |
| Homo sapiens | U14680 | AAA73985 | 1863aa |
| Bos taurus (cow) | AY077732 | AAL76094 | 1849aa |
| Pan troglodytes (chimpanzee) | AY365046 | AAR04849 | 1863aa |
| Canis familiaris | U50709 | AAC48663 | 1878aa |
| Monodelphis domestica | AY994160 | AAX92675 | 1844aa |
| Rattus norvegicus | AF036760 | AAC36493 | 1817aa |
| Mus musculus | U36475 | AAC52323 | 1812aa |
| Gorilla gorilla | AY589042 | AAT44835 | 1863aa |
| Pongo pygmaeus (Orangutan) | AY589040 | AAT44834 | 1863aa |
| Macaca mulatta (Rhesus monkey) | AY589041 | AAT44833 | 1863aa |
| BRCA2 ^†^ |  |  |  |
| Homo sapiens | U43746 | AAB07223 | 3418aa |
| Mus musculus | U89652 | AAB71377 | 3329aa |
| Rattus norvegicus | U89653 | AAB71378 | 3343aa |
| Felis catus | AB107955 | BAC75821 | 3372aa |
| Canis familiaris | AB043895 | BAB91245 | 3446aa |

^§^ For BRCA1, Felis catus sequence was excluded since the length of the protein sequence was only 948aa.

^†^ For BRCA2, Pan troglodytes and Bos taurus sequences were excluded since these are predicted sequences.
