# Supplementary material for: Evaluation of unclassified variants in the breast cancer susceptibility genes BRCA1 and BRCA2 using five methods: results from a population-based study of young breast cancer patients
Source: Breast Cancer Res. 2008 Feb 19;10(1):R19. doi: 10.1186/bcr1865 (PMC2374975; doi:10.1186/bcr1865)
Supplement: Additional file 3 — Word file containing a table listing all of the BRCA1/BRCA2 variants identified in this study along with our classification of each variant in comparison with the classification according to the BIC database. [file bcr1865-S3.doc]

Supplemental Table 2. Classification of significance of BRCA1/2 variants identified among early onset breast cancer patients in Los Angeles and comparison with classification according to BIC (Version updated April 05, 2007).

|  |  |  |  | UV classification |  |  |  |  |  | BIC classification |  |
| --- | --- | --- | --- | --- | --- | --- | --- | --- | --- | --- | --- |
| Variant | MAF in NHW (n=1105); HW (n=222);  AA (n=142) | Significance | Reason (references) | Frequency | Polyphen | Grantham score | Number of differences in alignment | Grantham/ Alignment |  | Mutation effect | Clinical significance |
| **BRCA1** |  |  |  |  |  |  |  |  |  |  |  |
| IVS1-115T>C | 0.3403; 0.3438; 0.2266 | Polymorphism | Intronic* |  |  |  |  |  |  | NA |  |
| Exon 2 |  |  |  |  |  |  |  |  |  |  |  |
| 185delAG | 0.0073; 0.0045; 0 | DDCV | Frameshift |  |  |  |  |  |  | Frameshift | Yes |
| IVS2-32delAT | 0; 0; 0.0035 | Polymorphism | Intronic* |  |  |  |  |  |  | NA |  |
| Exon 5 |  |  |  |  |  |  |  |  |  |  |  |
| Q60R | 0; 0.0023; 0 | UV |  | LFUV | Possible | 43 | 0 | Deleterious |  | NA |  |
| R71G | 0; 0.0068; 0 | DDCV | Splice variant, (1) |  | Probable | 125 | 0 | Deleterious |  | Missense | Yes |
| IVS5+1G>A | 0; 0.0023; 0 | DDCV | Splice variant |  |  |  |  |  |  | Splice | Yes |
| IVS5+21G>A | 0.0005; 0; 0 | Polymorphism | Intronic* |  |  |  |  |  |  | UV | Unknown |
| IVS5+23T>A | 0; 0; 0.0211 | Polymorphism | Intronic* |  |  |  |  |  |  | NA |  |
| IVS5-11T>G | 0.0009; 0; 0 | UV | Intronic, possibly creates a splice acceptor site near the end of intron 5 | LFUV |  |  |  |  |  | Splice | Yes |
| IVS7-34C/T | 0.2435; 0.1121; 0.0674 | Polymorphism | Intronic* |  |  |  |  |  |  | Polymorphism | No |
| Exon 8 |  |  |  |  |  |  |  |  |  |  |  |
| Y179C | 0; 0.0023; 0 | UV |  | LFUV | Probable | 194 | 0 | Deleterious |  | UV | Unknown |
| Exon 11 |  |  |  |  |  |  |  |  |  |  |  |
| L246V | 0.0014; 0; 0 | UV |  | LFUV | Benign | 32 | 1 | Neutral |  | UV | Unknown |
| 884G/A | 0; 0.0023; 0.0035 | Polymorphism | Synonymous |  |  |  |  |  |  | NA |  |
| 887G/A | 0; 0.0023; 0 | Polymorphism | Synonymous |  |  |  |  |  |  | NA |  |
| 943Ins10 | 0; 0; 0.0035 | DDCV | Frameshift |  |  |  |  |  |  | Frameshift | Yes |
| T276R | 0.0005; 0; 0 | UV |  | LFUV | Probable | 71 | 0 | Deleterious |  | UV | Unknown |
| 1100A/G | 0.0023; 0.0023; 0 | Polymorphism | Synonymous |  |  |  |  |  |  | Polymorphism | Unknown |
| S316G | 0.0005; 0; 0 | UV |  | LFUV | Possible | 56 | 0 | Deleterious |  | UV | Unknown |
| W321X | 0.0005; 0; 0 | DDCV | Nonsense |  |  |  |  |  |  | Nonsense | Yes |
| Q356R | 0.0682; 0.0251; 0.0106 | Polymorphism | (2-5) |  | Possible | 43 | 2 | Unknown |  | Polymorphism | Unknown |
| D369del(1225del3) | 0.0005; 0; 0 | UV | In frame deletion | LFUV |  |  |  |  |  | UV | Unknown |
| W372X | 0.0009; 0; 0 | DDCV | Nonsense |  |  |  |  |  |  | Nonsense | Yes |
| I379M | 0; 0; 0.0141 | UV |  | HFUV | Possible | 10 | 1 | Unknown |  | UV | No |
| S405P | 0.0005; 0; 0 | UV |  | LFUV | Possible | 74 | 2 | Unknown |  | NA |  |
| 1505delG | 0; 0.0023; 0 | DDCV | Frameshift |  |  |  |  |  |  | Frameshift | Yes |
| F486L | 0; 0.0023; 0 | UV |  | LFUV | Benign | 22 | 3 | Neutral |  | UV | Unknown |
| R496C | 0; 0.0023; 0 | UV |  | LFUV | Benign | 180 | 5 | Deleterious |  | UV | Unknown |
| R496H | 0.0005; 0; 0 | UV |  | LFUV | Benign | 29 | 5 | Neutral |  | UV | Unknown |
| N550H | 0; 0.0023; 0 | UV |  | LFUV | Benign | 68 | 3 | Unknown |  | UV | Unknown |
| Q563X | 0.0005; 0; 0 | DDCV | Nonsense |  |  |  |  |  |  | Nonsense | Yes |
| E597K | 0.0005; 0; 0 | UV |  | LFUV | Possible | 56 | 1 | Neutral |  | UV | Unknown |
| A622V | 0; 0.0023; 0 | UV |  | LFUV | Benign | 64 | 4 | Unknown |  | UV | Unknown |
| 2072delGAAA | 0.0005; 0; 0 | DDCV | Frameshift |  |  |  |  |  |  | Frameshift | Yes |
| 2090A/G | 0.0005; 0.0045; 0.0106 | Polymorphism | Synonymous |  |  |  |  |  |  | Polymorphism | Unknown |
| K679X | 0.0009; 0; 0 | DDCV | Nonsense |  |  |  |  |  |  | Nonsense | Yes |
| D693N | 0.0728; 0.0475; 0.0352 | UV |  | HFUV | Benign | 23 | 6 | Neutral |  | Polymorphism | No |
| 2201C/T | 0.3431; 0.3364; 0.2266 | Polymorphism | Synonymous |  |  |  |  |  |  | Polymorphism | No |
| T703T (2228A/G) | 0; 0.0023; 0.0035 | Polymorphism | Synonymous |  |  |  |  |  |  | UV | Unknown |
| N723D | 0; 0; 0.0035 | UV |  | LFUV | Benign | 23 | 2 | Unknown |  | UV | Unknown |
| E730X | 0.0005; 0; 0 | DDCV | Nonsense |  |  |  |  |  |  | Nonsense | Yes |
| R756K | 0; 0.0023; 0 | UV |  | LFUV | Benign | 26 | 4 | Neutral |  | NA |  |
| 2430T/C | 0.3393; 0.3257; 0.1950 | Polymorphism | Synonymous |  |  |  |  |  |  | Polymorphism | No |
| T790A | 0; 0; 0.0035 | UV |  | LFUV | Benign | 58 | 0 | Deleterious |  | UV | Unknown |
| 2576delC | 0.0005; 0; 0 | DDCV | Frameshift |  |  |  |  |  |  | Frameshift | Yes |
| K820E | 0; 0; 0.0282 | UV |  | HFUV | Benign | 56 | 4 | Neutral |  | UV | Unknown |
| 2594delC | 0.0009; 0; 0 | DDCV | Frameshift |  |  |  |  |  |  | Frameshift | Yes |
| R841W | 0.0009; 0.0023; 0 | UV |  | LFUV | Possible | 101 | 7 | Unknown |  | UV | Unknown |
| S868X | 0.0005; 0; 0 | DDCV | Nonsense |  |  |  |  |  |  | Nonsense | Yes |
| P871L | 0.3555; 0.3802; 0.7210 | Polymorphism | (2) |  | Benign | 98 | 9 | Neutral |  | Polymorphism | No |
| N877S | 0.0005; 0; 0 | UV |  | LFUV | Benign | 46 | 4 | Neutral |  | NA |  |
| 2852A/G | 0.0023; 0.0023; 0 | Polymorphism |  |  |  |  |  |  |  | NA |  |
| 2953delGTAinsC | 0.0005; 0; 0 | DDCV | Frameshift |  |  |  |  |  |  | Frameshift | Yes |
| S955X | 0.0005; 0; 0 | DDCV | Nonsense |  |  |  |  |  |  | Nonsense | Yes |
| 2994delA | 0.0005; 0; 0 | DDCV | Frameshift |  |  |  |  |  |  | NA |  |
| 2999C/T | 0.0005; 0; 0 | Polymorphism |  |  |  |  |  |  |  | NA |  |
| L965F | 0.0005; 0; 0 | UV |  | LFUV | Benign | 22 | 1 | Neutral |  | NA |  |
| M1008I | 0.0023; 0.0023; 0 | UV |  | LFUV | Benign | 10 | 5 | Neutral |  | UV | Unknown |
| E1038G | 0.3673; 0.3767; 0.2286 | Polymorphism | (3, 4) |  | Benign | 98 | 1 | Unknown |  | Polymorphism | No |
| S1040N | 0.0200; 0.0090; 0.0035 | UV |  | HFUV | Benign | 46 | 3 | Unknown |  | UV | Unknown |
| 3226delAG | 0.0005; 0; 0 | DDCV | Frameshift |  |  |  |  |  |  | NA |  |
| S1101N | 0.0005; 0; 0 | UV |  | LFUV | Benign | 46 | 1 | Unknown |  | UV | Unknown |
| S1140G | 0.0005; 0; 0.0211 | UV |  | HFUV | Benign | 56 | 4 | Neutral |  | UV | Unknown |
| 3481del11 | 0.0005; 0; 0 | DDCV | Frameshift |  |  |  |  |  |  | NA |  |
| K1183R | 0.3427; 0.3349; 0.2286 | Polymorphism | (3, 4) |  | Benign | 26 | 8 | Neutral |  | Polymorphism | No |
| S1187N | 0; 0.0023; 0 | UV |  | LFUV | Benign | 46 | 2 | Unknown |  | UV | Unknown |
| Q1200H | 0; 0; 0.0070 | UV |  | LFUV | Benign | 154 | 2 | Unknown |  | UV | No |
| R1203Q | 0.0005; 0; 0 | UV |  | LFUV | Benign | 43 | 5 | Neutral |  | UV | Unknown |
| 3788T/C | 0.0005; 0; 0 | Polymorphism | Synonymous |  |  |  |  |  |  | NA |  |
| N1236K | 0.0009; 0.0023; 0 | UV |  | LFUV | Possible | 94 | 3 | Unknown |  | UV | Unknown |
| P1238L | 0.0005; 0; 0 | UV |  | LFUV | Benign | 98 | 1 | Neutral |  | UV | Unknown |
| 3829delT | 0; 0.0023; 0 | DDCV | Frameshift |  |  |  |  |  |  | Frameshift | Yes |
| 3875del4 | 0; 0.0023; 0 | DDCV | Frameshift |  |  |  |  |  |  | Frameshift | Yes |
| N1272S | 0; 0.0023; 0 | UV |  | LFUV | Possible | 46 | 2 | Neutral |  | NA |  |
| Q1313X | 0.0005; 0; 0 | DDCV | Nonsense |  |  |  |  |  |  | Nonsense | Yes |
| R1347G | 0.0050; 0.0068; 0.0035 | UV |  | LFUV | Probable | 125 | 2 | Unknown |  | UV | Unknown |
| M1361L | 0.0005; 0; 0 | UV |  | LFUV | Benign | 15 | 7 | Unknown |  | UV | Unknown |
| 4184del4 | 0.0005; 0; 0 | DDCV | Frameshift |  |  |  |  |  |  | Frameshift |  |
| IVS11+36A>G | 0; 0; 0.0035 | UV | Intronic, possibly creates a splice donor site near the start of intron 11 | LFUV |  |  |  |  |  | NA |  |
| Exon 12 |  |  |  |  |  |  |  |  |  |  |  |
| 4232G/A | 0; 0.0023; 0.0035 | Polymorphism | Synonymous |  |  |  |  |  |  | NA |  |
| IVS12+9C>T | 0; 0.0023; 0 | UV | Intronic, possibly creates a splice donor site near the start of intron 12 | LFUV |  |  |  |  |  | UV | Unknown |
| Exon 13 |  |  |  |  |  |  |  |  |  |  |  |
| 4427T/C | 0.3419; 0.3303; 0.1979 | Polymorphism | Synonymous |  |  |  |  |  |  | Polymorphism | No |
| R1443X | 0.0005; 0; 0 | DDCV | Nonsense |  |  |  |  |  |  | Nonsense | Yes |
| Exon 15 |  |  |  |  |  |  |  |  |  |  |  |
| W1508X | 0; 0.0023; 0 | DDCV | Nonsense |  |  |  |  |  |  | Nonsense | Yes |
| S1512I | 0.0050; 0.0023; 0.0035 | Polymorphism | (6, 7) |  | Possible | 142 | 2 | Unknown |  | Polymorphism | No |
| V1534M | 0.0005; 0; 0.0035 | UV |  | LFUV | Possible | 21 | 5 | Neutral |  | UV | Unknown |
| Exon 16 |  |  |  |  |  |  |  |  |  |  |  |
| T1561I | 0; 0; 0.0035 | UV |  | LFUV | Possible | 89 | 1 | Unknown |  | UV | Unknown |
| Q1604Q (4931A/G) | 0.0009; 0; 0 | Polymorphism | Synonymous |  |  |  |  |  |  | UV | Unknown |
| S1613G | 0.3339; 0.3273; 0.2286 | Polymorphism | (3, 4) |  | Benign | 56 | 2 | Unknown |  | Polymorphism | No |
| M1628T | 0.0009; 0; 0 | UV |  | LFUV | Benign | 81 | 5 | Unknown |  | UV | Unknown |
| P1637L | 0.0005; 0; 0 | UV |  | LFUV | Probable | 98 | 1 | Unknown |  | UV | Unknown |
| M1652I | 0.0095; 0.0045; 0 | UV |  | LFUV | Probable | 10 | 2 | Unknown |  | UV | Unknown |
| IVS17+3A>G | 0.0005; 0; 0 | UV | Intronic, possibly creates a splice donor site near the start of intron 17 | LFUV |  |  |  |  |  | UV | Unknown |
| Exon 18 |  |  |  |  |  |  |  |  |  |  |  |
| A1708E | 0.0005; 0.0023; 0 | DDCV | (8-10) |  | Probable | 107 | 0 | Deleterious |  | Missense | Yes |
| 5254delG | 0.0005; 0; 0 | DDCV | Frameshift |  |  |  |  |  |  | NA |  |
| Exon 19 |  |  |  |  |  |  |  |  |  |  |  |
| T1720A | 0; 0.0023; 0 | UV |  | LFUV | Benign | 58 | 3 | Unknown |  | UV | Unknown |
| 5296del4 (5292del4) | 0.0005; 0; 0 | DDCV | Frameshift |  |  |  |  |  |  | Frameshift | Yes |
| R1726G | 0.0005; 0; 0 | UV |  | LFUV | Benign | 125 | 3 | Neutral |  | UV | Unknown |
| Exon 20 |  |  |  |  |  |  |  |  |  |  |  |
| R1751X | 0; 0; 0.0035 | DDCV | Nonsense |  |  |  |  |  |  | Nonsense | Yes |
| R1751L | 0.0005; 0; 0 | UV |  | LFUV | Probable | 102 | 0 | Deleterious |  | NA |  |
| 5382InsC | 0.0027; 0.0023; 0 | DDCV | Frameshift |  |  |  |  |  |  | Frameshift | Yes |
| Exon 21 |  |  |  |  |  |  |  |  |  |  |  |
| M1775R | 0; 0; 0.0035 | DDCV | (8, 10, 11) |  | Probable | 91 | 0 | Deleterious |  | Missense | Unknown |
| Exon 22 |  |  |  |  |  |  |  |  |  |  |  |
| M1783T | 0; 0; 0.0035 | UV |  | LFUV | Probable | 81 | 1 | Deleterious |  | UV | Unknown |
| C1787S, G1788D (CG1787>1788SD ) | 0; 0.0045; 0 | UV |  | LFUV | Probable | 112 | 0 | Deleterious |  | Missense | Unknown |
| W1782R | 0; 0; 0.0035 | UV |  | LFUV | Benign | 101 | 2 | Neutral |  | NA |  |
| W1782X | 0.0005; 0; 0 | DDCV | Nonsense |  |  |  |  |  |  | Nonsense | Yes |
|  |  |  |  |  |  |  |  |  |  |  |  |
| **BRCA2** |  |  |  |  |  |  |  |  |  |  |  |
| Exon 2 |  |  |  |  |  |  |  |  |  |  |  |
| 203G/A | 0.2642; 0.1923; 0.1127 | Polymorphism | Synonymous |  |  |  |  |  |  | Polymorphism | No |
| 218C>T | 0; 0; 0.0141 | Polymorphism | Synonymous |  |  |  |  |  |  | UV | Unknown |
| IVS2-7T>A | 0.0027; 0.0023; 0 | Polymorphism | Intronic* |  |  |  |  |  |  | UV | Unknown |
| Exon 3 |  |  |  |  |  |  |  |  |  |  |  |
| Y42C | 0.0023; 0.0023; 0 | UV |  | LFUV | Probable | 194 | 0 | Deleterious |  | UV | Unknown |
| P59A | 0; 0; 0.0035 | UV |  | LFUV | Probable | 27 | 0 | Deleterious |  | UV | Unknown |
| 426A/G | 0.0005; 0; 0 | Polymorphism | Synonymous |  |  |  |  |  |  | NA |  |
| T77A | 0; 0; 0.0035 | UV |  | LFUV | Benign | 58 | 0 | Deleterious |  | UV | Unknown |
| 460T/G | 0; 0; 0.0176 | Polymorphism | Synonymous |  |  |  |  |  |  | NA |  |
| Exon 7 |  |  |  |  |  |  |  |  |  |  |  |
| 746delG | 0; 0; 0.0035 | DDCV | Frameshift |  |  |  |  |  |  | Frameshift | Yes |
| R174H | 0.0005; 0; 0 | UV |  | LFUV | Benign | 29 | 4 | Neutral |  | UV | Unknown |
| IVS8-25T>C | 0.0005; 0; 0 | Polymorphism | Intronic* |  |  |  |  |  |  | NA |  |
| Exon 10 |  |  |  |  |  |  |  |  |  |  |  |
| N289H | 0.0341; 0.0826; 0.0322 | UV |  | HFUV | Possible | 68 | 0 | Deleterious |  | Polymorphism | No |
| S326R | 0.0005; 0.0023; 0 | UV |  | LFUV | Benign | 110 | 2 | Unknown |  | UV | No |
| Q347R | 0; 0; 0.0035 | UV |  | LFUV | Benign | 43 | 2 | Neutral |  | UV | Unknown |
| H372N (N372H) | 0.2784; 0.2964; 0.1151 | UV | Mixed association studies (12-14) | HFUV | Possible | 68 | 3 | Neutral |  | Polymorphism | No |
| P375S | 0.0005; 0; 0 | UV |  | LFUV | Probable | 74 | 0 | Deleterious |  | UV | Unknown |
| S384F | 0; 0.0023; 0 | UV |  | LFUV | Possible | 155 | 1 | Unknown |  | UV | No |
| W395G | 0; 0.0023; 0 | UV |  | LFUV | Probable | 184 | 2 | Unknown |  | UV | Unknown |
| 1503A/G | 0.0027; 0.0023; 0.0070 | Polymorphism | Synonymous |  |  |  |  |  |  | NA |  |
| 1593A/G | 0.0312; 0.0818; 0.0319 | Polymorphism | Synonymous |  |  |  |  |  |  | Polymorphism | No |
| E462G | 0.0005; 0; 0 | UV |  | LFUV | Possible | 98 | 0 | Deleterious |  | UV | Unknown |
| I505R | 0.0014; 0; 0 | UV |  | LFUV | Probable | 97 | 0 | Deleterious |  | NA |  |
| K513R | 0; 0.0023; 0 | UV |  | LFUV | Benign | 26 | 4 | Neutral |  | NA |  |
| D596H | 0.0005; 0; 0 | UV |  | LFUV | Possible | 81 | 0 | Deleterious |  | UV | No |
| 2016T/C | 0.0009; 0; 0 | Polymorphism | Synonymous |  |  |  |  |  |  | Polymorphism | Unknown |
| T598A | 0.0009; 0; 0 | UV |  | LFUV | Benign | 58 | 2 | Neutral |  | UV | No |
| G602R | 0.0005; 0; 0 | UV |  | LFUV | Probable | 125 | 0 | Deleterious |  | UV | Unknown |
| T630I | 0.0014; 0; 0 | UV |  | LFUV | Possible | 89 | 1 | Unknown |  | UV | Unknown |
| Exon 11 |  |  |  |  |  |  |  |  |  |  |  |
| 2139T/C | 0; 0; 0.0035 | UV | Second nucleotide of exon 11, possibly affect splicing | LFUV |  |  |  |  |  | NA |  |
| 2161insA | 0.0005; 0; 0 | DDCV | Frameshift |  |  |  |  |  |  | NA |  |
| 2166C/T | 0.0014; 0; 0 | Polymorphism | Synonymous |  |  |  |  |  |  | Polymorphism | No |
| P655R | 0.0036; 0; 0 | UV |  | LFUV | Probable | 103 | 0 | Deleterious |  | UV | Unknown |
| Q713L | 0; 0; 0.0035 | UV |  | LFUV | Possible | 113 | 2 | Unknown |  | UV | Unknown |
| Q742X | 0; 0.0023; 0 | DDCV | Nonsense |  |  |  |  |  |  | Nonsense | Yes |
| 2457T/C | 0.0346; 0.0891; 0.0326 | Polymorphism | Synonymous |  |  |  |  |  |  | UV | Unknown |
| K745E | 0; 0; 0.0035 | UV |  | LFUV | Benign | 56 | 4 | Neutral |  | NA |  |
| N900D | 0.0005; 0; 0 | UV |  | LFUV | Benign | 23 | 4 | Neutral |  | UV | Unknown |
| L929S | 0; 0; 0.0106 | UV |  | HFUV | Benign | 145 | 4 | Deleterious |  | UV | No |
| D935K | 0.0005; 0; 0 | UV |  | LFUV | Possible | 101 | 0 | Deleterious |  | NA |  |
| D935H | 0.0005; 0; 0 | UV |  | LFUV | Possible | 81 | 0 | Deleterious |  | UV | Unknown |
| K944X | 0; 0; 0.0035 | DDCV | Nonsense |  |  |  |  |  |  | Nonsense | Yes |
| 3034del4 | 0.0005; 0; 0 | DDCV | Frameshift |  |  |  |  |  |  | Frameshift | Yes |
| S976I | 0; 0; 0.0070 | UV |  | LFUV | Possible | 142 | 2 | Unknown |  | UV | No |
| I982M | 0; 0.0023; 0 | UV |  | LFUV | Benign | 10 | 4 | Neutral |  | UV | Unknown |
| 3185delA | 0.0005; 0; 0 | DDCV | Frameshift |  |  |  |  |  |  | NA |  |
| Q961Q (3111G/A) | 0.0018; 0; 0 | Polymorphism | Synonymous |  |  |  |  |  |  | UV | Unknown |
| N987I | 0; 0; 0.0106 | UV |  | HFUV | Probable | 149 | 2 | Deleterious |  | UV | No |
| N991D | 0.0355; 0.0863; 0.0471 | UV |  | HFUV | Benign | 23 | 4 | Neutral |  | UV | No |
| L1019V | 0; 0.0023; 0 | UV |  | LFUV | Benign | 32 | 0 | Deleterious |  | UV | Unknown |
| T1087I | 0.0005; 0; 0 | UV |  | LFUV | Benign | 89 | 3 | Unknown |  | NA |  |
| 3492insT | 0; 0.0023; 0 | DDCV | Frameshift |  |  |  |  |  |  | Frameshift | Yes |
| P1088P (3492T/C) | 0; 0; 0.0282 | Polymorphism | Synonymous |  |  |  |  |  |  | UV | Unknown |
| 3624A/G | 0.3150; 0.2123; 0.2210 | Polymorphism | Synonymous |  |  |  |  |  |  | Polymorphism | No |
| S1172S (3744G/A) | 0.0014; 0; 0 | Polymorphism | Synonymous |  |  |  |  |  |  | UV | Unknown |
| F1192C | 0; 0.0023; 0 | UV |  | LFUV | Probable | 205 | 2 | Unknown |  | UV | Unknown |
| 4017T/C | 0.1835; 0.1785; 0.2218 | Polymorphism | Synonymous |  |  |  |  |  |  | NA |  |
| C1290Y | 0; 0; 0.0035 | UV |  | LFUV | Probable | 194 | 1 | Neutral |  | UV | No |
| 4075delGT | 0.0009; 0; 0 | DDCV | Frameshift |  |  |  |  |  |  | Frameshift | Yes |
| L1356L (4296G/A) | 0.0023; 0.0023; 0 | Polymorphism | Synonymous |  |  |  |  |  |  | UV | Unknown |
| 1364L | 0; 0.0045; 0.0142 | UV |  | HFUV | Benign | 5 | 2 | Neutral |  | UV | Unknown |
| Q1396R | 0; 0; 0.0070 | UV |  | LFUV | Benign | 43 | 4 | Unknown |  | UV | Unknown |
| T1414M | 0; 0; 0.0106 | UV |  | HFUV | Benign | 81 | 4 | Neutral |  | UV | No |
| D1420Y | 0.0046; 0; 0 | UV |  | LFUV | Possible | 160 | 2 | Deleterious |  | Polymorphism | No |
| 4780delG | 0; 0; 0.0035 | DDCV | Frameshift |  |  |  |  |  |  | NA |  |
| 4791G/A | 0; 0.0023; 0.0607 | Polymorphism | Synonymous |  |  |  |  |  |  | Polymorphism | No |
| G1529R | 0.0009; 0; 0.0035 | UV |  | LFUV | Probable | 125 | 0 | Deleterious |  | UV | No |
| H1561N | 0; 0; 0.0141 | UV |  | HFUV | Probable | 68 | 1 | Unknown |  | UV | Unknown |
| T1566A | 0.0005; 0; 0 | UV |  | LFUV | Benign | 58 | 4 | Unknown |  | NA |  |
| V1610M | 0.0005; 0; 0 | UV |  | LFUV | Benign | 21 | 3 | Neutral |  | UV | Unknown |
| Y1672H | 0; 0.0023; 0 | UV |  | LFUV | Possible | 83 | 0 | Deleterious |  | NA |  |
| 5302InsA | 0; 0.0023; 0 | DDCV | Frameshift |  |  |  |  |  |  | Frameshift | Yes |
| S1733F | 0.0009; 0; 0.0035 | UV |  | LFUV | Possible | 155 | 1 | Unknown |  | UV | Unknown |
| 5427C/T | 0.0055; 0.0023; 0.0035 | Polymorphism | Synonymous |  |  |  |  |  |  | Polymorphism | No |
| 5580insA | 0.0005; 0; 0 | DDCV | Frameshift |  |  |  |  |  |  | NA |  |
| 5646A/G | 0; 0; 0.0319 | Polymorphism | Synonymous |  |  |  |  |  |  | NA |  |
| F1870X (5837TC>AG) | 0.0005; 0; 0 | DDCV | Nonsense |  |  |  |  |  |  | Nonsense | Yes |
| S1871N | 0; 0; 0.0035 | UV |  | LFUV | Benign | 46 | 1 | Neutral |  | UV | Unknown |
| N1880K | 0; 0; 0.0035 | UV |  | LFUV | Benign | 94 | 4 | Unknown |  | UV | Unknown |
| S1882X | 0.0005; 0; 0 | DDCV | Nonsense |  |  |  |  |  |  | Nonsense | Yes |
| D1902K | 0; 0; 0.0106 | UV |  | HFUV | Possible | 101 | 4 | Unknown |  | NA |  |
| 5946delCT | 0.0005; 0; 0 | DDCV | Frameshift |  |  |  |  |  |  | Frameshift | Yes |
| T1915M | 0.0234; 0.0068; 0.0036 | UV |  | HFUV | Benign | 81 | 4 | Unknown |  | UV | Unknown |
| D1923A | 0; 0; 0.0035 | UV |  | LFUV | Benign | 126 | 3 | Unknown |  | UV | Unknown |
| E1953X | 0.0005; 0; 0 | DDCV | Nonsense |  |  |  |  |  |  | Nonsense | Yes |
| C1960Y | 0; 0; 0.0035 | UV |  | LFUV | Probable | 194 | 4 | Unknown |  | UV | Unknown |
| L1965F | 0; 0.0023; 0 | UV |  | LFUV | Benign | 22 | 3 | Neutral |  | NA |  |
| H1966R | 0.0005; 0; 0 | UV |  | LFUV | Probable | 29 | 4 | Neutral |  | UV | Unknown |
| G1976V | 0; 0; 0.0035 | UV |  | LFUV | Probable | 109 | 0 | Deleterious |  | NA |  |
| 6174delT | 0.0027; 0; 0 | DDCV | Frameshift |  |  |  |  |  |  | Frameshift | Yes |
| I2033M | 0.0005; 0; 0 | UV |  | LFUV | Benign | 10 | 2 | Unknown |  | UV | Unknown |
| R2034C | 0.0041; 0.0023; 0 | UV |  | LFUV | Possible | 180 | 4 | Unknown |  | UV | Unknown |
| H2074N | 0; 0.0023; 0.0070 | UV |  | LFUV | Probable | 68 | 2 | Unknown |  | UV | Unknown |
| L2106P | 0.0005; 0; 0 | UV |  | LFUV | Benign | 98 | 3 | Neutral |  | UV | Unknown |
| R2108C | 0.0005; 0; 0 | UV |  | LFUV | Benign | 180 | 4 | Unknown |  | UV | Unknown |
| R2108H | 0.0009; 0; 0.0035 | UV |  | LFUV | Benign | 29 | 4 | Neutral |  | UV | Unknown |
| N2113S | 0.0005; 0; 0 | UV |  | LFUV | Benign | 46 | 4 | Unknown |  | UV | Unknown |
| H2116R | 0; 0; 0.0035 | UV |  | LFUV | Probable | 29 | 2 | Unknown |  | UV | No |
| V2138F | 0; 0; 0.0141 | UV |  | HFUV | Benign | 50 | 4 | Neutral |  | UV | Unknown |
| 6741C/G | 0.0005; 0.0023; 0.0568 | Polymorphism | Synonymous |  |  |  |  |  |  | Polymorphism | Unknown |
| 6872del4 | 0.0005; 0; 0 | DDCV | Frameshift |  |  |  |  |  |  | Frameshift | Yes |
| IVS11-73T>A | 0.0005; 0; 0 | UV | Intronic, possibly creates a splice acceptor site near the end of intron 11 | LFUV |  |  |  |  |  | NA |  |
| Exon 12 |  |  |  |  |  |  |  |  |  |  |  |
| I2285V | 0.0005; 0; 0 | UV |  | LFUV | Benign | 29 | 0 | Deleterious |  | UV | Unknown |
| IVS13+5G>C | 0.0005; 0; 0 | Polymorphism | Intronic* |  |  |  |  |  |  | UV | Unknown |
| Exon 14 |  |  |  |  |  |  |  |  |  |  |  |
| K2339N | 0; 0; 0.0282 | UV |  | HFUV | Benign | 94 | 1 | Unknown |  | UV | Unknown |
| E2340Q | 0; 0; 0.0035 | UV |  | LFUV | Benign | 29 | 1 | Unknown |  | NA |  |
| A2351T | 0.0005; 0; 0 | UV |  | LFUV | Benign | 58 | 2 | Unknown |  | UV | Unknown |
| 7297delCT | 0.0005; 0; 0 | DDCV | Frameshift |  |  |  |  |  |  | Frameshift | Yes |
| Q2384K | 0; 0; 0.0106 | UV |  | HFUV | Benign | 53 | 4 | Neutral |  | UV | Unknown |
| 7470A/G | 0.2201; 0.1697; 0.2043 | Polymorphism | Synonymous |  |  |  |  |  |  | Polymorphism | No |
| D2438H | 0.0005; 0; 0 | UV |  | LFUV | Possible | 81 | 2 | Unknown |  | NA |  |
| H2440R | 0; 0; 0.0352 | UV |  | HFUV | Probable | 29 | 3 | Neutral |  | UV | Unknown |
| A2466V | 0.0005; 0.0023; 0.0810 | UV |  | HFUV | Benign | 64 | 2 | Unknown |  | UV | Unknown |
| Exon 15 |  |  |  |  |  |  |  |  |  |  |  |
| I2490T | 0.0027; 0.0611; 0.0035 | UV |  | HFUV | Probable | 89 | 2 | Deleterious |  | UV | Unknown |
| R2494X | 0; 0.0023; 0 | DDCV | Nonsense |  |  |  |  |  |  | Nonsense | Yes |
| T2515I | 0.0009; 0; 0 | UV |  | LFUV | Possible | 89 | 2 | Unknown |  | UV | No |
| IVS16+6C>G | 0.0005; 0; 0.0106 | UV | Intronic, possibly creates a splice donor site near the start of intron 16 | HFUV |  |  |  |  |  | UV | Unknown |
| IVS16-14T/C | 0.4996; 0.5161; 0.5141 | Polymorphism | Intronic* |  |  |  |  |  |  | Polymorphism | No |
| Exon 17 |  |  |  |  |  |  |  |  |  |  |  |
| D2611A | 0; 0.0023; 0 | UV |  | LFUV | Probable | 126 | 0 | Deleterious |  | NA |  |
| V2620I | 0.0005; 0; 0 | UV |  | LFUV | Benign | 29 | 0 | Deleterious |  | NA |  |
| 8138del5 | 0.0005; 0; 0 | DDCV | Frameshift |  |  |  |  |  |  | Frameshift | Yes |
| Exon 18 |  |  |  |  |  |  |  |  |  |  |  |
| R2678G | 0; 0; 0.0035 | UV |  | LFUV | Probable | 125 | 0 | Deleterious |  | UV | Unknown |
| A2717S | 0.0018; 0; 0 | UV |  | LFUV | Benign | 99 | 4 | Unknown |  | UV | No |
| V2728I | 0.0036; 0; 0 | UV |  | LFUV | Benign | 29 | 2 | Neutral |  | Polymorphism | No |
| RLTVG2743del (8457del15) | 0.0005; 0; 0 | UV | In frame deletion | LFUV |  |  |  |  |  | UV | Unknown |
| Exon 19 |  |  |  |  |  |  |  |  |  |  |  |
| 8651delTTTTCInsA | 0.0005; 0; 0 | DDCV | Frameshift |  |  |  |  |  |  | NA |  |
| V2820V (8688A/C) | 0; 0; 0.0070 | Polymorphism | Synonymous |  |  |  |  |  |  | UV | Unknown |
| Exon 20 |  |  |  |  |  |  |  |  |  |  |  |
| S2835P | 0; 0; 0.0035 | UV |  | LFUV | Benign | 74 | 4 | Neutral |  | UV | No |
| R2842H | 0.0005; 0; 0 | UV |  | LFUV | Possible | 29 | 0 | Deleterious |  | UV | Unknown |
| 8761delAG (8762delGA) | 0.0005; 0; 0 | DDCV | Frameshift |  |  |  |  |  |  | Frameshift | Yes |
| E2856A | 0.0032; 0; 0 | UV |  | LFUV | Possible | 107 | 0 | Deleterious |  | UV | No |
| Q2859X | 0.0005; 0; 0 | DDCV | Nonsense |  |  |  |  |  |  | Nonsense | Yes |
| 8823insT | 0; 0.0023; 0 | DDCV | Frameshift |  |  |  |  |  |  | NA |  |
| Exon 21 |  |  |  |  |  |  |  |  |  |  |  |
| V2908G | 0.0005; 0; 0 | UV |  | LFUV | Possible | 109 | 0 | Deleterious |  | UV | Unknown |
| IVS21+4A>G | 0.0005; 0; 0 | Polymorphism | Intronic* |  |  |  |  |  |  | UV | Unknown |
| IVS21-1G>A | 0.0005; 0; 0 | DDCV | Splice variant |  |  |  |  |  |  | Splice | Yes |
| Exon 22 |  |  |  |  |  |  |  |  |  |  |  |
| I2944F | 0; 0.0068; 0.0211 | UV |  | HFUV | Benign | 21 | 0 | Deleterious |  | UV | Unknown |
| K2950N | 0.0009; 0.0023; 0.0035 | UV |  | LFUV | Benign | 94 | 0 | Deleterious |  | UV | Unknown |
| A2951T | 0.0032; 0.0361; 0 | Polymorphism | (6) |  | Benign | 58 | 0 | Deleterious |  | Polymorphism | No |
| V2969M | 0.0005; 0; 0 | UV |  | LFUV | Benign | 21 | 0 | Deleterious |  | UV | Unknown |
| R2973C | 0.0005; 0; 0 | UV |  | LFUV | Probable | 180 | 0 | Deleterious |  | UV | Unknown |
| 9168insA | 0.0005; 0; 0 | DDCV | Frameshift |  |  |  |  |  |  | Frameshift | Yes |
| IVS22+26del9 | 0; 0; 0.0035 | Polymorphism | Intronic* |  |  |  |  |  |  | NA |  |
| Exon 23 |  |  |  |  |  |  |  |  |  |  |  |
| E3002K | 0.0005; 0; 0 | UV |  | LFUV | Benign | 56 | 0 | Deleterious |  | UV | Unknown |
| T3013I | 0.0014; 0; 0 | UV |  | LFUV | Benign | 89 | 3 | Unknown |  | UV | No |
| A3029T | 0.0005; 0; 0 | UV |  | LFUV | Benign | 58 | 0 | Deleterious |  | UV | Unknown |
| P3039P (9345G/A) | 0.0009; 0; 0 | DDCV | Splice variant, (15) |  |  |  |  |  |  | Splice | Yes |
| Exon 24 |  |  |  |  |  |  |  |  |  |  |  |
| R3052W | 0; 0.0023; 0 | UV |  | LFUV | Probable | 101 | 0 | Deleterious |  | UV | Unknown |
| V3079I | 0; 0; 0.0070 | UV |  | LFUV | Benign | 29 | 0 | Deleterious |  | UV | Unknown |
| Exon 25 |  |  |  |  |  |  |  |  |  |  |  |
| Y3092C | 0; 0.0023; 0 | UV |  | LFUV | Probable | 194 | 0 | Deleterious |  | UV | Unknown |
| Y3098H | 0.0005; 0.0023; 0 | UV |  | LFUV | Benign | 83 | 4 | Neutral |  | UV | Unknown |
| Exon 26 |  |  |  |  |  |  |  |  |  |  |  |
| G3212R | 0; 0; 0.0106 | UV |  | LFUV | Possible | 125 | 0 | Deleterious |  | UV | Unknown |
| Exon 27 |  |  |  |  |  |  |  |  |  |  |  |
| N3221T | 0.0005; 0; 0 | UV |  | LFUV | Benign | 65 | 2 | Neutral |  | NA |  |
| V3244I | 0; 0; 0.0282 | UV |  | HFUV | Benign | 29 | 4 | Neutral |  | UV | Unknown |
| K3326X | 0.0091; 0.0068; 0 | Polymorphism | (16) |  |  |  |  |  |  | Polymorphism | No |
| R3370R (10338G/A) | 0.0018; 0.0023; 0 | Polymorphism | Synonymous |  |  |  |  |  |  | UV | Unknown |
| T3374I | 0; 0; 0.0035 | UV |  | LFUV | Benign | 89 | 2 | Unknown |  | UV | Unknown |
| I3412V | 0.0028; 0.0622; 0.0958 | Polymorphism | Located closer to the end of the gene than the polymorphic nonsense mutation K3326X, probably disposable |  | UNKNOWN | 29 | 4 | Neutral |  | UV | Unknown |

Abbreviations: MAF: Minor allele frequency; NHW: non-Hispanic whites, HW: Hispanic whites, AA: African-Americans. DDCV: definitely-disease causing variant; UV: unclassified variants; LFUV: low-frequency UV ; HFUV: high frequency UV; NA: not applicable (never been reported in BIC).

* Intronic variant, unlikely to influence splicing.

**References**

1. Vega, A., Campos, B., Bressac-De-Paillerets, B., Bond, P. M., Janin, N., Douglas, F. S., Domenech, M., Baena, M., Pericay, C., Alonso, C., Carracedo, A., Baiget, M., and Diez, O. The R71G BRCA1 is a founder Spanish mutation and leads to aberrant splicing of the transcript. Hum Mutat, *17:* 520-521, 2001.

2. Dunning, A. M., Chiano, M., Smith, N. R., Dearden, J., Gore, M., Oakes, S., Wilson, C., Stratton, M., Peto, J., Easton, D., Clayton, D., and Ponder, B. A. Common BRCA1 variants and susceptibility to breast and ovarian cancer in the general population. Hum Mol Genet, *6:* 285-289, 1997.

3. Durocher, F., Shattuck-Eidens, D., McClure, M., Labrie, F., Skolnick, M. H., Goldgar, D. E., and Simard, J. Comparison of BRCA1 polymorphisms, rare sequence variants and/or missense mutations in unaffected and breast/ovarian cancer populations. Hum Mol Genet, *5:* 835-842, 1996.

4. Friedman, L. S., Ostermeyer, E. A., Szabo, C. I., Dowd, P., Lynch, E. D., Rowell, S. E., and King, M. C. Confirmation of BRCA1 by analysis of germline mutations linked to breast and ovarian cancer in ten families. Nat Genet, *8:* 399-404, 1994.

5. Tavtigian, S. V., Deffenbaugh, A. M., Yin, L., Judkins, T., Scholl, T., Samollow, P. B., de Silva, D., Zharkikh, A., and Thomas, A. Comprehensive statistical study of 452 BRCA1 missense substitutions with classification of eight recurrent substitutions as neutral. J Med Genet, *43:* 295-305, 2006.

6. Deffenbaugh, A. M., Frank, T. S., Hoffman, M., Cannon-Albright, L., and Neuhausen, S. L. Characterization of common BRCA1 and BRCA2 variants. Genet Test, *6:* 119-121, 2002.

7. Phelan, C. M., Dapic, V., Tice, B., Favis, R., Kwan, E., Barany, F., Manoukian, S., Radice, P., van der Luijt, R. B., van Nesselrooij, B. P., Chenevix-Trench, G., kConFab, Caldes, T., de la Hoya, M., Lindquist, S., Tavtigian, S. V., Goldgar, D., Borg, A., Narod, S. A., and Monteiro, A. N. Classification of BRCA1 missense variants of unknown clinical significance. J Med Genet, *42:* 138-146, 2005.

8. Chapman, M. S. and Verma, I. M. Transcriptional activation by BRCA1. Nature, *382:* 678-679, 1996.

9. Vallon-Christersson, J., Cayanan, C., Haraldsson, K., Loman, N., Bergthorsson, J. T., Brondum-Nielsen, K., Gerdes, A. M., Moller, P., Kristoffersson, U., Olsson, H., Borg, A., and Monteiro, A. N. Functional analysis of BRCA1 C-terminal missense mutations identified in breast and ovarian cancer families. Hum Mol Genet, *10:* 353-360, 2001.

10. Carvalho, M. A., Marsillac, S. M., Karchin, R., Manoukian, S., Grist, S., Swaby, R. F., Urmenyi, T. P., Rondinelli, E., Silva, R., Gayol, L., Baumbach, L., Sutphen, R., Pickard-Brzosowicz, J. L., Nathanson, K. L., Sali, A., Goldgar, D., Couch, F. J., Radice, P., and Monteiro, A. N. Determination of Cancer Risk Associated with Germ Line BRCA1 Missense Variants by Functional Analysis. Cancer Res, *67:* 1494-1501, 2007.

11. Williams, R. S. and Glover, J. N. M. Structural Consequences of a Cancer-causing BRCA1-BRCT Missense Mutation. J. Biol. Chem., *278:* 2630-2635, 2003.

12. Spurdle, A. B., Hopper, J. L., Chen, X., Dite, G. S., Cui, J., McCredie, M. R., Giles, G. G., Ellis-Steinborner, S., Venter, D. J., Newman, B., Southey, M. C., and Chenevix-Trench, G. The BRCA2 372 HH genotype is associated with risk of breast cancer in Australian women under age 60 years. Cancer Epidemiol Biomarkers Prev, *11:* 413-416, 2002.

13. Healey, C. S., Dunning, A. M., Teare, M. D., Chase, D., Parker, L., Burn, J., Chang-Claude, J., Mannermaa, A., Kataja, V., Huntsman, D. G., Pharoah, P. D., Luben, R. N., Easton, D. F., and Ponder, B. A. A common variant in BRCA2 is associated with both breast cancer risk and prenatal viability. Nat Genet, *26:* 362-364, 2000.

14. Breast Cancer Association, C. Commonly studied single-nucleotide polymorphisms and breast cancer: results from the Breast Cancer Association Consortium. J Natl Cancer Inst, *98:* 1382-1396, 2006.

15. Peelen, T., van Vliet, M., Bosch, A., Bignell, G., Vasen, H. F., Klijn, J. G., Meijers-Heijboer, H., Stratton, M., van Ommen, G. J., Cornelisse, C. J., and Devilee, P. Screening for BRCA2 mutations in 81 Dutch breast-ovarian cancer families. Br J Cancer, *82:* 151-156, 2000.

16. Mazoyer, S., Dunning, A. M., Serova, O., Dearden, J., Puget, N., Healey, C. S., Gayther, S. A., Mangion, J., Stratton, M. R., Lynch, H. T., Goldgar, D. E., Ponder, B. A., and Lenoir, G. M. A polymorphic stop codon in BRCA2. Nat Genet, *14:* 253-254, 1996.
